# Thalamic activations in rat brain by fMRI during tactile (forepaw, whisker) and non-tactile (visual, olfactory) sensory stimulations

**DOI:** 10.1371/journal.pone.0267916

**Published:** 2022-05-06

**Authors:** Basavaraju G. Sanganahalli, Garth J. Thompson, Maxime Parent, Justus V. Verhagen, Hal Blumenfeld, Peter Herman, Fahmeed Hyder

**Affiliations:** 1 Magnetic Resonance Research Center (MRRC), Yale University, New Haven, Connecticut, United States of America; 2 Department of Radiology and Biomedical Imaging, Yale University, New Haven, Connecticut, United States of America; 3 iHuman Institute, ShanghaiTech University, Shanghai, China; 4 The John B. Pierce Laboratory, New Haven, Connecticut, United States of America; 5 Department of Neuroscience, Yale University, New Haven, Connecticut, United States of America; 6 Department of Neurology, Yale University, New Haven, Connecticut, United States of America; 7 Department of Neurosurgery, Yale University, New Haven, Connecticut, United States of America; 8 Department of Biomedical Engineering, Yale University, New Haven, Connecticut, United States of America; University of North Carolina at Chapel Hill, UNITED STATES

## Abstract

The thalamus is a crucial subcortical hub that impacts cortical activity. Tracing experiments in animals and post-mortem humans suggest rich morphological specificity of the thalamus. Very few studies reported rodent thalamic activations by functional MRI (fMRI) as compared to cortical activations for different sensory stimuli. Here, we show different portions of the rat thalamus in response to tactile (forepaw, whisker) and non-tactile (visual, olfactory) sensory stimuli with high field fMRI (11.7T) using a custom-build quadrature surface coil to capture high sensitivity signals from superficial and deep brain regions simultaneously. Results demonstrate reproducible thalamic activations during both tactile and non-tactile stimuli. Forepaw and whisker stimuli activated broader regions within the thalamus: ventral posterior lateral (VPL), ventral posterior medial (VPM), lateral posterior mediorostral (LPMR) and posterior medial (POm) thalamic nuclei. Visual stimuli activated dorsal lateral geniculate nucleus (DLG) of the thalamus but also parts of the superior/inferior colliculus, whereas olfactory stimuli activated specifically the mediodorsal nucleus of the thalamus (MDT). BOLD activations in LGN and MDT were much stronger than in VPL, VPM, LPMR and POm. These fMRI-based thalamic activations suggest that forepaw and whisker (i.e., tactile) stimuli engage VPL, VPM, LPMR and POm whereas visual and olfactory (i.e., non-tactile) stimuli, respectively, recruit DLG and MDT exclusively.

## Introduction

The thalamus is the major relay to the cerebral cortex. In the somatosensory, visual and auditory systems, the connectivity of feedback projections onto thalamic neurons is linked to the tuning preferences of cortical cells [1–3]. The thalamus has been described as the gate to the cortex, encompassing well defined, functionally and anatomically separated nuclei. The thalamus contains more than 30 nuclei, each connecting to diverse cortical areas [4–6]. Since these cortical-subcortical circuits can be affected by various brain disorders [7–10], functional parcellation of thalamic nuclei is of particular interest. The exact role of thalamus in cognitive functions has not been studied in detail. Research has shown that selective damage to thalamic nuclei is involved in various neurological [11] and psychiatric disorders [12], therefore thalamus may also be linked in non-sensory functions [13].

Tactile (forepaw, whisker) sensory activations, produce excitatory inputs on thalamocortical neurons of the ventrobasal complex through lemniscal projections [14–17] and indirect inhibitory inputs through the thalamic reticular nucleus [18]. The balance between excitatory and inhibitory inputs therefore shapes the responses of thalamocortical neurons to tactile stimuli. Previous electrophysiological and functional MRI (fMRI) experiments have observed the thalamic activations during tactile stimulations distributed in different thalamic nuclei: VPL, VPM, LPMR and POm regions [19–27].

In the visual system the retinal axons from both eyes come together to form the optic nerves enroute to reaching the brain. From there, fibers are known to project to the ventral and dorsal lateral geniculate nuclei (ventral LGN and dorsal LGN, respectively), lateral posterior nucleus, pretectum, and superior colliculus (SC) [28–31].

The olfactory sensory pathway is unique compared to other senses since this sensory information does not get relayed through thalamus before it reaches the cortex [32–35]. Olfactory connections with the mediodorsal thalamus (MDT) do exist, however, especially from pyriform cortex (PCX) and to the orbitofrontal cortex (OFC), forming the PCX-MDT-OFC pathway and therefore the "olfactory thalamus" (MDT) acts as a higher-order thalamic relay and not as primary sensory relay [36–39]. The role of the MDT is accordingly thought to mediate more subtle functions such as difficult odor discriminations, odor preferences and odor reversal learning [37, 40, 41]. The MDT may further play a role in attention to odor [42, 43], as assessed by human fMRI [43, 44]. Electrophysiological evidence points to rather narrowly tuned odor representations in the MDT and beta oscillations-based coherent coupling with the PCX [45]. We are not aware of studies demonstrating fMRI-based odor responses in the rodent MDT, which are important to study brain-wide functional connectivity of the MDT in various states and behaviors.

Studies by other groups, including ours, have demonstrated the use of fMRI to study brain function in response to different sensory paradigms in anesthetized rodents [23, 24, 26, 27, 29–31, 46–63]. Most of these studies reported changes in the cortical fMRI activity and only few studies have demonstrated activations in the subcortical regions (thalamus, superior/inferior colliculus and the basal ganglia).

fMRI activations of subcortical regions are harder to distinguish compared to cortical activity because of lower sensitivity from the radio-frequency (RF) coil placed on the top of the rodent’s head. In the rat brain, most subcortical regions are several millimeter deeper than the cerebral cortex and sensory stimuli activate small subcortical areas (tenths of mm^3^) than cortical regions (several mm^3^). Interpretation of subcortical activations at lower signal to noise ratio (SNR) and insufficient spatial resolution is challenging. Currently with the availability of higher field strength, better shimming, higher spatial resolution and SNR we can obtain both cortical and sub-cortical fMRI activity. A recent study using ultra high magnetic field (15.2T) showed clear cortical and thalamic responses to somatosensory stimulation in mouse under ketamine-xylazine anesthesia [23]. They also compared these activities in terms of magnetic field strength dependency (9.4T vs 15.2T). At ultra-high field they found a significantly higher SNR in the somatosensory cortex, thalamus and the secondary somatosensory area. The evoked blood-oxygenation level dependent (BOLD) responses in the somatosensory cortex and thalamus were much more reproducible at 15.2T, as compared to 9.4T [23]. Similar reproducible somatosensory forelimb, whisker barrel and thalamic activations were observed in the same rat preparation under α-chloralose anesthesia at high magnetic field 11.7T [25, 26, 62]. A recent study employed diffusion-weighted fMRI to explore the rat thalamocortical pathway [10]. This study demonstrated the sensitivity of diffusion-weighted fMRI over traditional spin-echo (SE) BOLD signals in detecting the localized thalamic activity during somatosensory stimulation. All these studies used ultra-high field scanner and high SNR for the reproducible subcortical activations. In our studies, we used a custom-built quadrature surface coil to improve SNR of cortical and subcortical regions at 11.7T. In cortical studies the first choice is to use a surface coil because of its high local sensitivity. Although it is possible to see thalamic activation, it is not reproducible because of the distance from the coil. Therefore, we improved the sensitivity of the measurement (i.e. increase the SNR at subcortical regions) by building a quadrature surface coil. When we compared the SNR from surface coil to quadrature coil, we observed uniform SNR at subcortical regions (S1 Fig). By using quadrature coil design, we observed reproducible cortical and thalamic activations in all the sensory paradigms we studied. In view of this methodological advance, the purpose of our study was to map out the functionally active portions of the rat thalamus for different sensory (forepaw, whisker, visual and olfactory) stimuli by high field fMRI (11.7T). Overall, this study comprehensively demonstrated reproducible cortical and thalamic fMRI BOLD responses across different sensory paradigms in rat brain.

## Materials and methods

### Animal preparation

All animal procedures were approved by the Institutional Animal Care and Use Committee (Yale University School of Medicine: Hyder-11194). All procedures complied with the regulations of the Animals (scientific procedures) Act 1986 and reporting follows the ARRIVE (animal research: reporting of in vivo experiments) guidelines. Adult male Sprague-Dawley and Long-Evans rats (Charles River, Wilmington, MA; fed ad libitum, 180-350g) were used. Blood gas tensions (pCO2, pO2, pH) (35±5 mmHg, 110±14 mmHg, 7.34±0.04), blood pressure (110±26 mmHg) and the core body temperature (37 ± 1°C) measured from artificially ventilated (70% N2O:30% O2) animals were within the normal range. Animal preparation for imaging experiments were described in detail in our previous studies [25, 57, 64, 65].

Animal preparation for forepaw, whisker, visual and olfactory studies: For forepaw and whisker stimulations we used Sprague-Dawley rats (Forepaw *n* = 8, whisker *n = 8*). Sprague-Dawley rats were anesthetized with isoflurane (2–2.5%) during surgery (tracheostomy, arterial and venous catheter). Intraperitoneal injection of α-chloralose with a bolus of 80 mg/kg and supplemental 40 mg/kg/h was used for the maintenance [66]. Isoflurane was discontinued after 15–20 minutes of α-chloralose injection. For visual studies we used Long-Evans rats (*n* = 8) and anesthesia was maintained under urethane (intraperitoneal injection of 1.5g/kg B.W with additional doses 0.13g/kg) [29]. For olfactory studies we used spontaneously breathing Sprague- Dawley rats (*n = 8*) and anesthesia was maintained with urethane as explained above and in our previous studies [67, 68]. We collected at least five trials in each subject and sensory modality. The data were averaged across trials and across subjects. Based on our prior observations eight subjects in each sensory modality was expected to provide sufficient statistical power.

### fMRI experiments

Details of fMRI measurements are discussed elsewhere [25, 57, 64]. We used a Bruker horizontal scanner (11.7T, Bruker AVANCE, Billerica, MA) using a custom-built quadrature radiofrequency coil (32 mm in diameter). We applied a FASTMAP approach for the local shimming (half line width of water < 15–20 Hz). Anatomical images were obtained using a RARE contrast sequence with a minimum spatial resolution of 200×200×1000 μm. We used a single shot GE-EPI (TR:1000 ms, TE:15ms) for the forepaw, whisker and visual fMRI experiments with a spatial resolution of 400×400×1000 μm. We collected the data from 5 slices covering somatosensory region for forepaw and whisker studies and visual part of the cortex for the visual stimulation experiments [29, 57, 64, 69]. Olfactory studies were performed using single shot GE-EPI imaging sequence and covered a larger part of the brain including the olfactory bulb (OB) with similar imaging parameters described above (TR = 1000 ms; TE = 15 ms; number of slices = 20; slice thickness = 500 μm, slice gap = 500 μm).

### Stimulus presentation

The detailed description of stimulus delivery for all of the sensory (forepaw, whisker, visual and olfactory) stimulation paradigms can be found in our earlier work [56, 66–68].

#### Forepaw stimulation

Electrical stimulation of the forepaw was achieved by inserting two copper needles between 2^nd^ and 4^th^ digits of the forepaw using a custom written Spike2 script which was controlled by μ1401 A-D converter unit (CED, Cambridge, UK). We used a block stimulus paradigm with 30s rest and 30s stimulation (2mA, 3Hz, 0.3ms, Square pulse) [64, 66].

#### Whisker stimulation

Naturalistic stimulation of whiskers by air puff was achieved using a custom-built whisker stimulator. The details are described in our earlier studies [56, 66, 69]. We used an ON-OFF pattern of 30-second pre-stimulus baseline, followed by 30-second whisker deflection, followed by 60-second post stimulus [66, 69].

#### Visual stimulation

Visual stimulus delivery system is described in our earlier work [29, 56]. A standard block stimulation protocol (30s off and 30s on) was used in all experiments. Bilateral visual stimulation was achieved by targeting blue LED light flashes (1Hz, 50 ms).

#### Olfactory stimulation

Odor delivery was achieved through a custom-built flow-dilution olfactometer. The details of the olfactometer and odor delivery procedures are described in our previous studies [56, 67, 70, 71]. Odors were delivered in a block design paradigm (60s off, 60s on, 120s off). We tested three different types of odorants (isoamyl-acetate (IAA), ethyl-butyrate (EB) and methyl-valerate (MV) at 20% concentration). These ester molecule odors with fruit-like smells produce similar but not identical activation patterns [67, 68, 71].

### Data analysis and statistics

We used custom MATLAB code integrated with Bioimage Suite (Yale School of Medicine, 2015, bioimagesuite.yale.edu) and SPM 12 (The FIL Methods group, 2015, www.fil.ion.ucl.ac.uk/spm/software/spm12). The details of co-registration, bias field correction, slice time correction, motion correction and postprocessing were described in detail in our earlier work [25, 64, 67, 70, 71]. Brain anatomical images from each animal were subsequently registered to the study template brain using a nonlinear registration (50 iterations, normalized mutual information, and otherwise default). Functional images were co-registered between rats after slice-timing correction followed by motion-correction (to the middle volume rather than the first volume) using SPM12 (The FIL Methods group, 2015, www.fil.ion.ucl.ac.uk/spm/software/spm12). Subsequently, the corresponding animal’s nonlinear transformation to the study template brain was applied to the functional images using the BioImage Suite software. To facilitate inter-animal comparisons, data was blurred in every slice using a Gaussian filter (σ = 2 voxels/0.250mm, size = 8 voxels/1mm). We generated BOLD activation maps using T-test between baseline and stimulated image volumes corrected for multiple comparison with p<0.05. We used sequential goodness of fit metatest (SGoF) [72] for statistical significance (p<0.05) corrected for multiple comparisons. BOLD activation T-maps were overlaid on to the corresponding anatomical slices. The time courses of BOLD activation (from individual subjects) were averaged across grouped subjects and represented as averaged across many subjects. Mean ± standard deviation (SD) was used to represent the BOLD responses.

## Results

**Fig 1:**
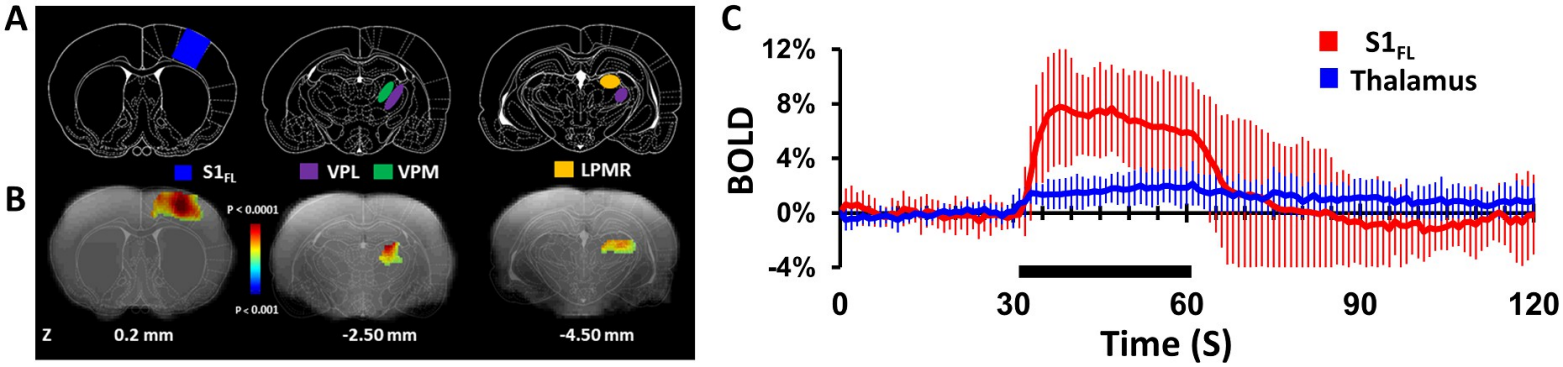
Forepaw stimulation-induced activation of rat somatosensory cortex and thalamus revealed by high-field (11.7T) fMRI (8 rats, 28 trials). Significant BOLD activations were observed at the contralateral primary somatosensory cortex area of forelimb (S1_FL_) and thalamic activations were found in different parts of ventral posterior lateral (VPL), ventral posterior medial (VPM), lateral posterior thalamic nucleus mediorostral (LPMR) during left forepaw stimulation (2 mA, 0.3 ms, 3 Hz). BOLD activation maps, displayed as colored statistical maps (a 2-sample, unpaired, T-test with equal variance and one tail for stimulation (30s) > baseline (30s)) overlaid on the corresponding anatomical images. **A** and **B** represents coronal slices corresponding to cortical and thalamic regions positioned at 0.2 mm anterior to bregma for S1_FL_, 2.50 and 4.50 mm posterior to bregma for VPL, VPM and LPMR regions. To correlate activations in cortical and thalamic regions, reference stereotaxic atlas images were overlaid on the corresponding anatomical images. **C** represents the mean ± SD time course of BOLD activations at S1_FL_ and thalamic (VPL, VPM and LPMR) regions.

Significant BOLD activations were observed at the primary somatosensory cortex (S1_FL_) and thalamus (Fig 1) during forepaw stimulation. Different parts of thalamic nuclei including VPL, VPM and LPMR regions were observed. Fig 1A represents coronal slices corresponding to cortical and thalamic regions positioned at S1_FL_, VPL, VPM and LPMR regions. The BOLD activation maps in Fig 1B are averaged data from eight rats (24 trials) for S1_FL_ and thalamic activations registered to anatomical space. To correlate activations in cortical and thalamic regions, reference stereotaxic atlas images were overlaid on the corresponding anatomical images. The mean ± SD of BOLD time courses across all rats from the S1_FL_ and VPL during 30 s of forepaw stimulation are shown in Fig 1C. The observed cortical (S1_FL_) and thalamic (VPL) BOLD responses are consistent with previous studies [24–26, 64, 73]. The magnitude of averaged BOLD responses (30s) was larger at S1_FL_ (6.3±1.8%) than thalamic (1.6±0.3%) regions. We observed reproducible BOLD responses in the S1_FL_ and thalamus across trials and across subjects. Repeated trials in the same subject and across subjects produced activations in approximately the same locations (S2 Fig).

**Fig 2:**
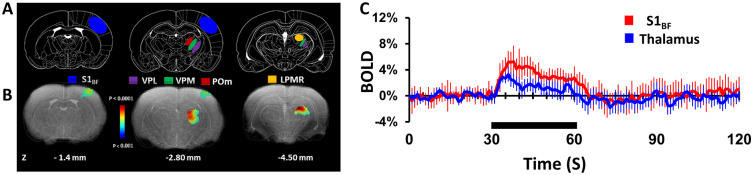
Whisker stimulation-induced activation of rat somatosensory cortex and thalamus revealed by high-field (11.7T) fMRI (8 rats, 22 trials). Significant contralateral BOLD activations were observed at the primary somatosensory barrel cortex (S1_BF_) and thalamic activations were found in different parts of ventral posterior lateral (VPL), ventral posterior medial (VPM), lateral posterior thalamic nucleus mediorostral (LPMR) and posterior medial (POm) during left whisker stimulation by air puff (22 whiskers, 2 mm, 3 Hz). BOLD activation maps, displayed as colored statistical maps (a 2-sample, unpaired, T-test with equal variance and one tail for stimulation (30s) > baseline (30s)) overlaid on the corresponding anatomical images. **A** and **B** represents coronal slices corresponding to cortical and thalamic regions positioned at 1.4 mm posterior to bregma for S1_BF_, 2.80 and 4.50 mm posterior to bregma for VPL, VPM and LPMR regions. To correlate activations in cortical and thalamic regions, reference stereotaxic atlas images were overlaid on the corresponding anatomical images. **C** represents the mean ± SD time course of BOLD activations at S1_BF_ and thalamic regions (VPL, VPM, LPMR and POm).

Whisker stimulation evoked significant BOLD activity in the somatosensory whisker barrel field (S1_BF_) and various thalamic nuclei as shown in Fig 2. Fig 2A and 2B illustrates group activation maps displayed as an overlay registered onto a common anatomical space. This included S1_BF_ which is slightly posterior to the location of S1_FL_, whereas ventral posterior medial (VPM), lateral posterior mediorostral (LPMR) and posterior medial thalamus (POm) are quite like those thalamic areas activated by forepaw stimuli (Fig 1). Trial to trial S1_BF_ and thalamic BOLD responses in the same subject as well as across other subjects are shown in S3 Fig. The BOLD responses were observed only in the contralateral S1_BF_ and the ventrobasal complex of thalamus and were similar to our previous observations [26, 56, 66, 69, 74, 75], because ipsilateral whiskers were shaved to remove spurious artifacts. Fig 2C shows averaged BOLD responses from eight rats (24 trials) from the contralateral S1_BF_ (3.3±1.1%) and thalamic nuclei (1.4±0.8%).

**Fig 3:**
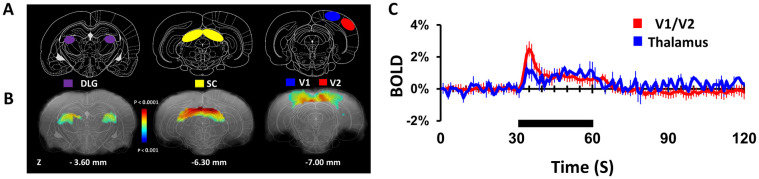
Visual stimulation-induced activation of rat visual cortex and thalamus revealed by high-field (11.7T) fMRI (8 rats, 21 trials). Significant BOLD activations were observed at the primary and secondary visual cortices (V1/V2), superior colliculus (SC) and thalamic activations were mainly found in dorsal parts of the lateral geniculate nucleus (DLG) during bilateral eye stimulation (1Hz, 50 ms, Blue light). BOLD activation maps, displayed as colored statistical maps (a 2-sample, unpaired, T-test with equal variance and one tail for stimulation (30s) > baseline (30s)) overlaid on the corresponding anatomical images. **A** and **B** represents coronal slices corresponding to visual cortices, superior colliculus (SC) and dorsal lateral geniculate nucleus (DLG) of the thalamus positioned at 7.0 mm posterior to bregma for V1/V2, 6.30 and 3.60 mm posterior to bregma for SC and DLG regions. To correlate activations in cortical and thalamic regions, reference stereotaxic atlas images were overlaid on the corresponding anatomical images. **C** represents the mean ± SD time course of BOLD activations at V1/V2 and DLG regions.

Visual stimulation evoked significant BOLD activations in the primary (V1) and secondary (V2) visual cortex, superior colliculus (SC) and thalamic regions like lateral geniculate nucleus (DLG) of the thalamus (Fig 3). Both eyes stimulated with blue light (1Hz, 50 ms) with equal intensity (30 lux) evoked significant and reproducible BOLD activations. BOLD activation maps observed at V1/V2 (7 mm posterior to bregma) and DLG (~3–5 mm posterior to bregma) matched well with the visual pathway as described in rat brain atlas [76] and are in good agreements with prior results [29–31]. Reproducible BOLD activations were seen across trials and subjects in DLG, V1/V2 and the dorsal layers of the SC (S4 Fig). The average V1/V2 and DLG response time courses are shown in Fig 3C. The average BOLD response magnitudes at V1/V2 and DLG are 0.92 ± 0.05% and 0.82 ± 0.04% respectively. However, the V1/V2 initial peak was significantly (p<0.0014) larger than DLG peak height.

**Fig 4:**
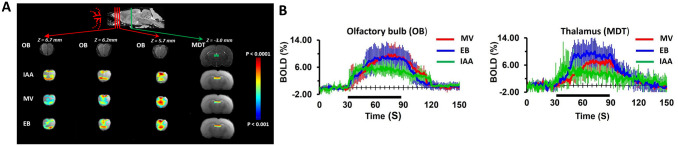
Odor stimulation-induced activation of rat Olfactory Bulb (OB) and thalamus revealed by high-field (11.7T) fMRI (8 rats, 24 trials). To correlate activations in OB and thalamic regions, reference stereotaxic atlas images were overlaid on the corresponding anatomical images shown in the top row (**A**). BOLD activations were observed in the OB and medial dorsal thalamic nuclei (MDT) during different odors stimulation (IAA: isoamyl acetate, MV: methyl valerate, EB: ethyl butyrate. BOLD activation maps were calculated during 1 minute odor stimulation. BOLD activation maps, displayed as colored statistical maps (a 2-sample, unpaired, T-test with equal variance and one tail for stimulation (60s) > baseline (30s)) overlaid on the corresponding anatomical images. Coronal slices corresponding to OB are positioned 6.7 mm, 6.2 mm, 5.7 mm anterior to bregma and 3 mm posterior to bregma for MDT. **B**: The mean ± SD time course of BOLD activations at OB and MDT regions for different odors were shown.

BOLD activity was simultaneously measured in olfactory bulb (OB) and the brain (including mediodorsal thalamic nucleus (MDT) and higher olfactory structures). Since we are interested in comparing responses from OB and thalamus, activation maps for other higher (including cortical) olfactory regions are not shown here. Fig 4A shows BOLD activations maps in the OB and MDT during different odors stimuli (IAA: isoamyl-acetate, MV: methyl-valerate, EB: ethyl-butyrate) averaged across eight rats. MDT responses to different odors were highly reproducible, and to our knowledge this is the first fMRI study in rodents to show this thalamic activation. We compared the temporal dynamics of BOLD signals during odor stimulation. Fig 4B shows the time courses of BOLD activations from OB and MDT. All three odors displayed similar responses in OB and MDT. The mean BOLD signal changes at the OB for IAA, MV and EB were 5.0 ±1.06%, 6.85 ±2.56% and 6.90 ±2.41% respectively. Mean BOLD signal changes at MDT for IAA, MV and EB are 3.62 ±1.0%, 4.5 ±2.6% and 6.98 ±3.1% respectively. We observed highly reproducible OB and MDT activations across different trials and subjects. Odor evoked bulbar BOLD responses are similar to our previous studies [67, 68, 71].

The volume of BOLD activation in the whole brain (cortex, bulb, and thalamus) during forepaw, whisker, visual and olfactory stimuli were 37.5 mm^3^, 16.16 mm^3^, 63.58 mm^3^ and 106.96 mm^3^. The spatial area was found to be larger in olfactory stimulation since the whole bulb and thalamic areas were included. Some regions overlapped across forepaw and whisker stimulation in the cortex (16.52 mm^3^) and thalamus (10.91 mm^3^).

Preliminary results of this study were presented in abstract form at ISMRM (https://cds.ismrm.org/protected/17MProceedings/PDFfiles/5351.html) and BRAIN & BRAIN PET 2017 [58] conference proceedings.

## Discussion

Here we demonstrate a clear modality-specific cortical and thalamic activation in response to tactile (forepaw, whisker) and non-tactile (visual, olfactory) stimuli. Our results revealed robust BOLD changes in both cortex and thalamic regions. Forepaw and whisker stimuli activated predominantly VPL and VPM, visual stimuli activated dorsal LGN and olfactory stimuli activated MDT. The thalamus is involved in many functions of visual, auditory, gustatory, and somatosensory senses, such as sensory gating and attention modulation [4]. Olfactory processing is unique where the first level of information is not passing through thalamus. Olfactory thalamus was not given much attention in understanding the olfactory function and the exact role of MDT in olfaction is poorly understood [32]. Here, we provide first fMRI evidence in a rodent model that implicates the MDT in olfactory processing. In contrast to most prior fMRI studies which used surface RF coils (some used volume coils also), we used a quadrature coil system that sums the two signals from each coil with a 90° phase difference. Thus, the quadrature coil we used gives a √2 boost in SNR compared to a single RF coil of the same dimension.

### Thalamic responses to tactile stimulation

During tactile stimulation (forepaw, whisker) VPL, VPM, POm and LPMR thalamic nuclei were strongly activated (Figs 1 and 2). Tactile stimuli provide excitatory inputs on thalamocortical neurons of the ventrobasal complex (VPL and VPM/POm) through lemniscal projections [15]. In our previous studies [25] we have shown using electrophysiological measurements ~4 ms delay in neural responses between VPL and S1_FL_, which is consistent with other findings that show similar magnitude/latency response between cortex and thalamus activity to forepaw, hindpaw, and whisker stimuli [16].

The BOLD responses at somatosensory forelimb and whisker barrel area observed in individual subjects as well as group averaged maps matched well with standard atlas [76]. Activation in thalamic regions was largely distributed in VPL, VPM, LPMR and POm regions. We found a significant difference in the magnitudes of the BOLD responses between S1_FL_, S1_BF_ and thalamic regions (Figs 1B and 2B). These regional differences in BOLD results are consistent with previous studies [24, 25, 27, 64, 69]. Given that the magnitude of BOLD response depends on the degree of uncoupling between changes in blood flow (CBF) and oxygen consumption (CMR_O2_), a smaller BOLD response in thalamic vs. cortical regions may not indicate smaller neuronal activity per se. Previously we showed that despite a much smaller BOLD response in the VPL, the metabolic demand between cortex and thalamus are quite similar [25]. The relationship between neural activity to the evoked blood flow or metabolic changes in cortical areas [57, 60, 77] as well as in non-cortical structures such as thalamus, hippocampus, basal ganglia, and brainstem [78, 79] indicate that neurovascular couplings regionally different, contributing to the regional variability of the BOLD response, similar to what we observe in our study. This coupling mainly depends on cell types, cell densities and unreactive vasculature or poor blood supply which would result in the demand for nutrients exceeding the supply [80]. To understand the BOLD signals, one must know how several cell types in the brain (excitatory neurons, inhibitory neurons, astrocytes) and vascular cells (pericytes, vascular smooth muscle and endothelial cells), and their modulation by ascending projection neurons contribute to both metabolism and hemodynamic changes. The thalamus consists of three basic cell types (relay cells, interneurons, and reticular cells) that are involved in thalamic processing. Relay cells, which relay information from the sensory input to the cortex, are glutamatergic (i.e., use glutamate as a neurotransmitter). Interneurons are GABAergic (i.e., use GABA, or γ-aminobutyric acid, as a neurotransmitter), and their projections are limited locally to their dorsal thalamic site of origin. The thalamo-reticular neurons are located between the thalamus and the cortex and represents an ideal hub for corticothalamic communications. It receives excitatory projections from thalamocortical and corticothalamic neurons and sends inhibitory efferents to all nuclei of the dorsal thalamus. Regional differences in BOLD signals should also reflect our increasing understanding of how neurons, astrocytes and vascular cells can be differentially affected and have correspondingly different effects on the BOLD signal.

Only few studies have been successful in using ultra high field fMRI to study the whole somatosensory pathway in both mice and rats under different anesthetic conditions [23, 26]. Prior studies using high field and high SNR were also successfully observed thalamic activations during both forepaw and whisker stimulations [24, 27]. The thalamocortical pathway in rodent somatosensory system was also mapped by using diffusion-weighted fMRI [10]. These studies demonstrate the sensitivity of diffusion-weighted fMRI over traditional gradient echo BOLD in detecting the thalamic activations at the same field strength.

The basic mechanisms underlying neurovascular coupling in BOLD activations require simultaneous multimodal fMRI, electrophysiological / optical imaging recordings. Previous studies from rat model of epilepsy [79, 81] observed negative BOLD in caudate-putamen or hippocampus in spite of a large increase in neural activity during seizures cautioning in interpreting BOLD signals in physiology and pathology from these regions. In a few of the trials and subjects we also observed bilateral thalamic responses to unilateral tactile stimuli. Some other previous studies also noted bilateral BOLD activation in thalamus during forepaw stimulations [82].

### Thalamic responses to non-tactile stimulation

During visual stimuli our fMRI responses (with multiple slices) enabled reproducible measurement of BOLD activations from multiple foci (V1/V2 and DLG) (Fig 3). In the rodent visual system photosensitive rods and cones activated by light from external environment connected to retinal ganglion cells through the layers of retina transmit information (intensity, color, spatial pattern) to the brain. We observed very reliable and reproducible single trial BOLD responses in both V1/V2 and DLG during bilateral stimulation with blue light. In previous studies we and others have observed differential evoked BOLD responses to different colors of light (white, blue, green and red) [29, 30].

Significant BOLD activations of whole OB and higher olfactory structures including the mediodorsal thalamic nucleus (MDT) were observed (Fig 4). MDT responses to different odors were highly reproducible, and this is the first olfactory fMRI study in rodents to show thalamic activation. It confirms prior electrophysiological MDT odor responses in rodents (for summary, see [45]) and complements human MDT fMRI studies [43, 44].

Among all senses olfaction is unique in terms of sensory processing. The first olfactory processing occurs at the glomerular region of OB and followed into higher olfactory regions such as PCX, OFC and thalamic nuclei (MDT). The subtle yet important roles of the MDT in olfaction are emerging study of interest (see e.g., [37, 42]). Rodent fMRI of the MDT and associated brain structures may become a valuable approach toward understanding the MDTs role in olfactory perception and odor-guided behaviors.

### Limitations of the current study

fMRI experiments in rodents require use anesthesia for subject immobility during the scan. We used different anesthetics for different sensory paradigms. Anesthetics affect neuronal activity and influence neurovascular/neurometabolic coupling. The type and depth of anesthesia influences the BOLD responses in different sensory modalities [51]. In earlier studies we had optimized stimulus parameters to obtain robust BOLD responses to tactile (forepaw: 2mA, 3Hz; whisker: 2mm 3 Hz) [25, 56, 66, 69] and non-tactile (visual: 1Hz blue light; olfactory: 20% odor) [29, 70] stimulation. We also tried different stimulation frequencies to check whether cortex and thalamus have different frequency tuning curves [83]. Thalamic responses reported in the literature during sensory stimulations were different across different anesthetized states (e.g. α-chloralose vs medetomidine) [24, 25, 84]. In our studies we used α-chloralose for forepaw and whisker sensory modalities, whereas visual and olfactory studies were performed under urethane anesthesia. This was chosen based on our prior imaging experience of different tactile and non-tactile sensory paradigms [56] to achieve the most reliable responses in the animal models. We obtained a reproducible and reliable fMRI BOLD response to both forepaw and whisker stimulation under α-chloralose anesthesia; however, the evoked BOLD responses were not reliable for visual and olfactory responses. Urethane and medetomidine anesthesia were very much suitable for reproducible visual and olfactory sensory experiments based on our prior experience. This is also in consistent with published rodent fMRI literature [29–31, 56]. Also, following the same logic to get the most reliable responses we used different rat strains for different sensory experiments. We used Sprague-Dawley for the forepaw, whisker, olfaction experiments and Long-Evans for visual sensory experiments, since the pigmented Long Evans rats have better visual acuity as compared to albino Sprague-Dawley rats [85–87]. Despite these weaknesses of different rat strain, anesthetics, the simultaneous observation of cortical and thalamic activities suggests that the thalamic parcellation across these different sensory modalities could be used for future studies.

Different cortical/thalamic networks are involved in triggering various kinds of BOLD signals include excitatory neurons, mixed neuronal populations, astroglia, and axonal tracts. Importantly, it is not clear which kinds of activity are capable of triggering BOLD responses, placing limitations on interpretation for scientific applications. fMRI BOLD activation maps are thought to reflect the underlying spatial layout of neural activity (LFP and MUA). While neural activity maps contained reliable structure at the sub-millimeter scale, fMRI maps of object selectivity contained information at larger scales.

## Conclusion

Some of the inconsistencies for thalamic activations observed in literature are due to differential anesthetic effects or low BOLD sensitivity (SNR) in the thalamus. Here we tried to reproducibly map cortical and thalamic activations at high field 11.7T for different sensory modalities using a quadrature surface coil with improved SNR as compared to traditional surface coil. Future studies combining high-resolution neuroanatomy (e.g., DTI) can provide the morphological basis of the high-resolution functional parcellation of the rat thalamus. Diffusion-weighted fMRI has emerged as a promising approach for overcoming some of BOLD limitations for the subcortical regions [10, 88], but this comes at the cost of lower SNR compared to BOLD data. While our data shows sufficient differentiation of thalamic nuclei with different sensory stimuli, we can still improve further the specificity of the thalamic measurements. The use of two separate quadrature surface coils (one dorsally and other ventrally) can further improve SNR while also providing better brain coverage. Similarly, slightly smaller sized coils of the quadrature system would also boost SNR more than the current conditions. While parallel imaging using multiple RF coils would boost SNR dramatically more, the subcortical regions would be sacrificed. In summary, these types of experiments should provide further insights into the interactions between cortical and thalamic areas and provide a mechanistic basis to understand thalamo-cortical activity.

## Supporting information

S1 Fig Signal to noise (SNR) comparison test was performed. Single shot gradient echo planar (GE-EPI) images obtained with 400×400 μm in-plane spatial resolution with a temporal resolution of 1s in rat brain with 2mm thickness. Data was collected using surface coil (**A**) and quadrature coil (**B**). The SNR is ubiquitously high in cortical and subcortical regions using a quadrature coil as compared to surface coil. **C** and **D** shows the pictures of custom-built surface coil and the quadrature coil.
(PDF)

S2 Fig BOLD responses from the contralateral forelimb area (S1FL) and thalamic regions during 3 Hz forepaw stimulation (0.3 ms pulses; 2 mA) in Sprague-Dawley rats. Reproducibility of S1FL and thalamic BOLD activation maps in the same subject during left paw stimulations (see Trial column; left (**A**)) as well as other subjects (see Rat column; right (**B**)). The statistical t maps were generated by comparison of the mean signals from 30 s baseline and stimulation periods. All data shown are from single trial runs. Reproducibility was quantitatively assessed across different trials using dice similarity coefficient (DSC). DSC were above 0.5 across all trials.
(PDF)

S3 Fig BOLD responses from the contralateral somatosensory whisker barrel area (S1BF) and thalamic regions during 3 Hz whisker stimulation (22 whiskers, 2mm) in Sprague-Dawley rats. Reproducibility of S1BF and thalamic BOLD activation maps in the same subject during left whisker stimulations (see Trial column; left (**A**)) as well as other subjects (see Rat column; right (**B**)). The statistical t maps were generated by comparison of the mean signals from 30 s baseline and stimulation periods. All data shown are from single trial runs. Reproducibility was quantitatively assessed across different trials using dice similarity coefficient (DSC). DSC were above 0.5 across all trials.
(PDF)

S4 Fig BOLD responses from the visual cortex (V1 and V2), superior colliculus (SC) and thalamic (dLGN) regions during 1 Hz bilateral visual stimulation (50 ms, Blue light) in Long-Evans rats. Reproducibility of BOLD activation maps in the same subject during bilateral visual stimulations (see Trial column; left (**A**)) as well as other subjects (see Rat column; right (B)). The statistical t maps were generated by comparison of the mean signals from 30 s baseline and stimulation periods. All data shown are from single trial runs. Reproducibility was quantitatively assessed across different trials using dice similarity coefficient (DSC). DSC were above 0.5 across all trials.
(PDF)
