# Supplementary material for: Thalamic activations in rat brain by fMRI during tactile (forepaw, whisker) and non-tactile (visual, olfactory) sensory stimulations
Source: PLoS One. 2022 May 6;17(5):e0267916. doi: 10.1371/journal.pone.0267916 (PMC9075615; doi:10.1371/journal.pone.0267916)
Supplement: S1 Fig — Single shot gradient echo planar (GE-EPI) images obtained with 400×400 μm in-plane spatial resolution with a temporal resolution of 1s in rat brain with 2mm thickness. Data was collected using surface coil (A) and quadrature coil (B). The SNR is ubiquitously high in cortical and subcortical regions using a quadrature coil as compared to surface coil. C and D shows the pictures of custom-built surface coil and the quadrature coil. (PDF) [file pone.0267916.s001.pdf]

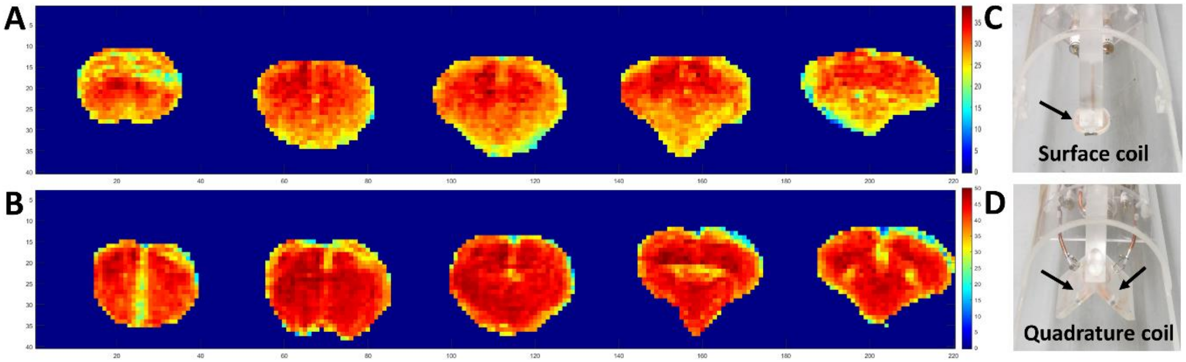

S1 Fig. Signal to noise (SNR) comparison test was performed. Single shot gradient echo planar (GE-EPI) images obtained with 400×400 μm in-plane spatial resolution with a temporal resolution of 1s in rat brain with 2mm thickness. Data was collected using surface coil (**A**) and quadrature coil (**B**). The SNR is ubiquitously high in cortical and subcortical regions using a quadrature coil as compared to surface coil. **C** and **D** shows the pictures of custom-built surface coil and the quadrature coil
