# Supplementary material for: Thalamic activations in rat brain by fMRI during tactile (forepaw, whisker) and non-tactile (visual, olfactory) sensory stimulations
Source: PLoS One. 2022 May 6;17(5):e0267916. doi: 10.1371/journal.pone.0267916 (PMC9075615; doi:10.1371/journal.pone.0267916)
Supplement: S3 Fig — Reproducibility of S1BF and thalamic BOLD activation maps in the same subject during left whisker stimulations (see Trial column; left (A)) as well as other subjects (see Rat column; right (B)). The statistical t maps were generated by comparison of the mean signals from 30 s baseline and stimulation periods. All data shown are from single trial runs. Reproducibility was quantitatively assessed across different trials using dice similarity coefficient (DSC). DSC were above 0.5 across all trials. (PDF) [file pone.0267916.s003.pdf]

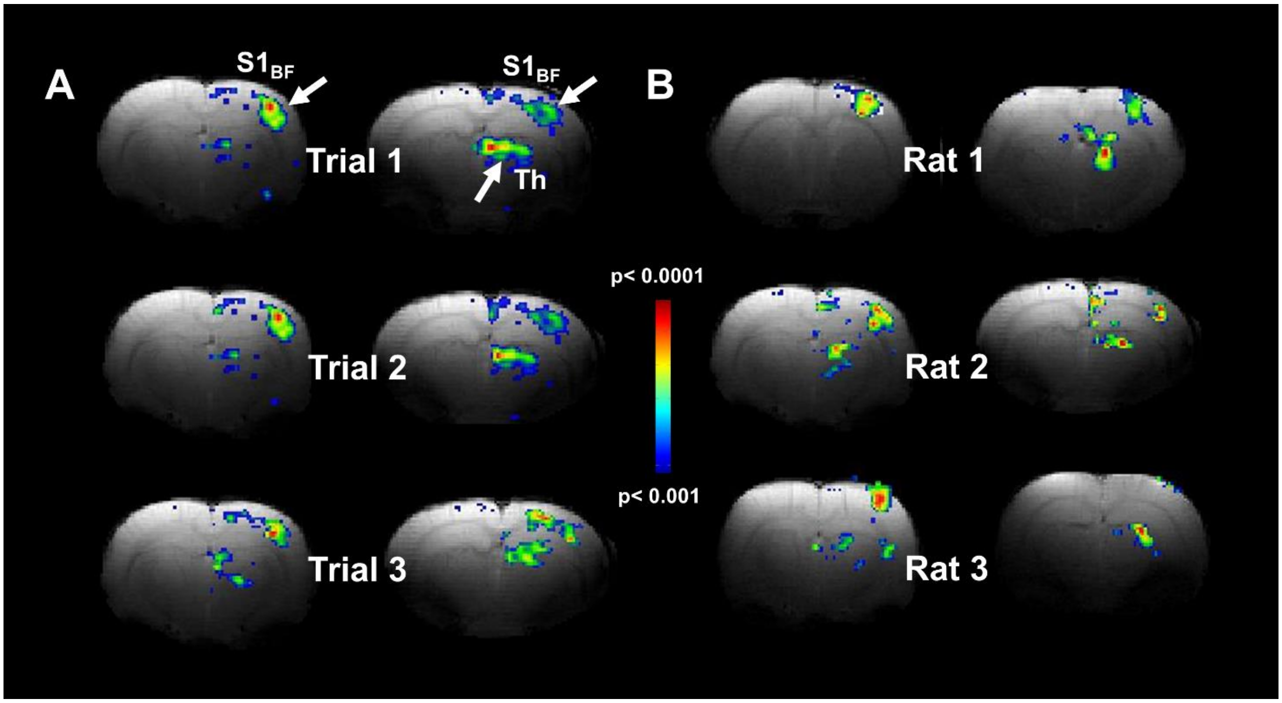

S3 Fig. BOLD responses from the contralateral somatosensory whisker barrel area ($S1_{BF}$) and thalamic regions during 3 Hz whisker stimulation (22 whiskers, 2mm) in Sprague-Dawley rats. Reproducibility of $S1_{BF}$ and thalamic BOLD activation maps in the same subject during left whisker stimulations (see Trial column; left (**A**)) as well as other subjects (see Rat column; right (**B**)). The statistical t maps were generated by comparison of the mean signals from 30 s baseline and stimulation periods. All data shown are from single trial runs. Reproducibility was quantitatively assessed across different trials using dice similarity coefficient (DSC). DSC were above 0.5 across all trials.
