# Supplementary material for: Thalamic activations in rat brain by fMRI during tactile (forepaw, whisker) and non-tactile (visual, olfactory) sensory stimulations
Source: PLoS One. 2022 May 6;17(5):e0267916. doi: 10.1371/journal.pone.0267916 (PMC9075615; doi:10.1371/journal.pone.0267916)
Supplement: S4 Fig — Reproducibility of BOLD activation maps in the same subject during bilateral visual stimulations (see Trial column; left (A)) as well as other subjects (see Rat column; right (B)). The statistical t maps were generated by comparison of the mean signals from 30 s baseline and stimulation periods. All data shown are from single trial runs. Reproducibility was quantitatively assessed across different trials using dice similarity coefficient (DSC). DSC were above 0.5 across all trials. (PDF) [file pone.0267916.s004.pdf]

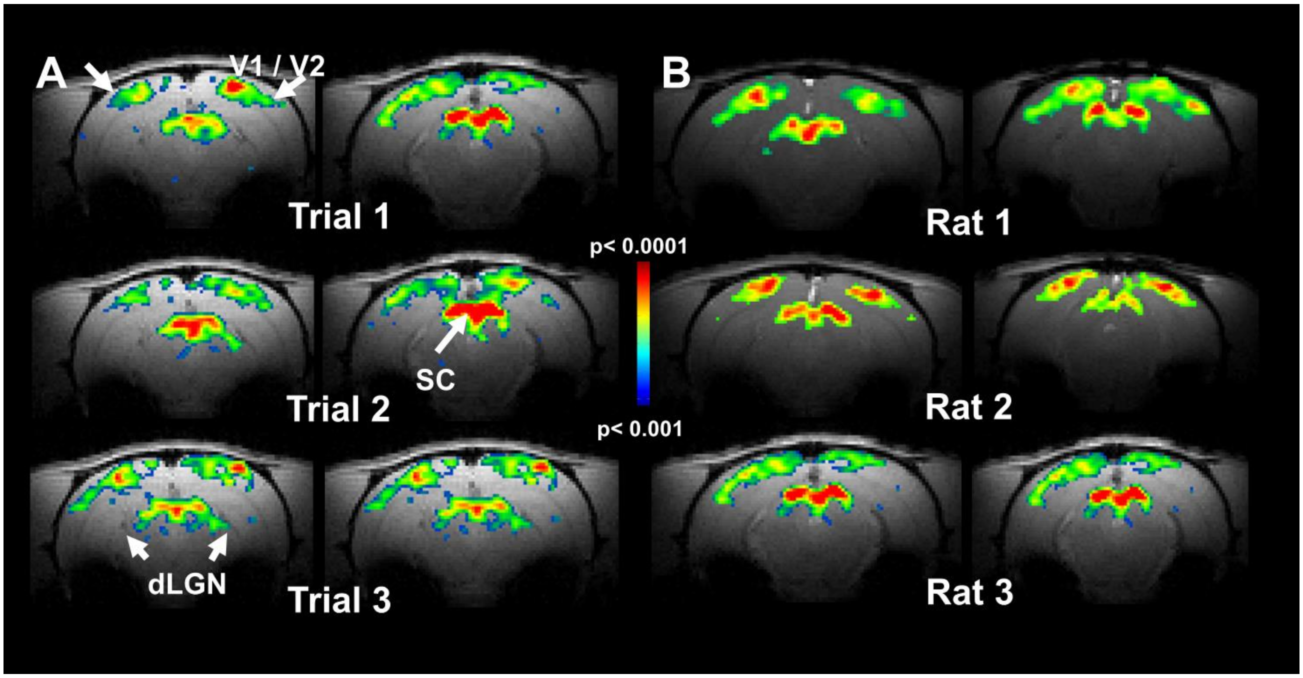

S4 Fig. BOLD responses from the visual cortex (V1 and V2), superior colliculus (SC) and thalamic (dLGN) regions during 1 Hz bilateral visual stimulation (50 ms, Blue light) in Long-Evans rats. Reproducibility of BOLD activation maps in the same subject during bilateral visual stimulations (see Trial column; left (**A**)) as well as other subjects (see Rat column; right (B)). The statistical t maps were generated by comparison of the mean signals from 30 s baseline and stimulation periods. All data shown are from single trial runs. Reproducibility was quantitatively assessed across different trials using dice similarity coefficient (DSC). DSC were above 0.5 across all trials.
